# Media-Induced and Psychological Factors That Foster Empathy Through Virtual Reality in Nursing Education: 2×2 Between-Subjects Experimental Study

**DOI:** 10.2196/59083

**Published:** 2025-03-31

**Authors:** Kuo-Ting Huang, Zexin Ma, Lan Yao

**Affiliations:** 1 Department of Information Culture and Data Stewardship, University of Pittsburgh, 135 N Bellefield Ave, Room 616, Pittsburgh, PA, 15213, United States, 1 4123839941; 2 Department of Communication, University of Connecticut, Storrs, CT, United States; 3 School of Nursing, Oakland University, Rochester, MI, United States

**Keywords:** nursing education, narrative transportation, presence, virtual reality, game-based learning, affective empathy

## Abstract

**Background:**

Virtual reality (VR) has emerged as a promising tool in medical education, particularly for fostering critical skills such as empathy. However, how VR, combined with perspective-taking, influences affective empathy in nursing education remains underexplored.

**Objective:**

This study investigates the influence of VR and perspective-taking on affective empathy in nursing education, focusing on 4 psychological factors: perceived self-location, narrative transportation, emotional engagement, and affective empathy.

**Methods:**

A 2×2 between-subjects design was used, involving 69 nursing undergraduates from two Midwest universities. The participants engaged with a narrative-focused video game, That Dragon, Cancer, in either VR or non-VR conditions and from the perspective of either parents or clinicians.

**Results:**

VR significantly enhanced perceived self-location (*P*=.01), while adopting a clinician’s perspective amplified emotional engagement (*P*=.03). However, VR did not significantly influence narrative transportation (*P*=.35). An interaction effect was found between the platform and player’s perspective on narrative transportation (*P*=.04). Several indirect effects of media elements on affective empathy were observed via other psychological factors, though the direct effect of VR on affective empathy was not significant (*P*=.84).

**Conclusions:**

These findings underscore the potential of VR in medical education, suggesting that perspective-taking should be carefully considered when designing immersive learning experiences. The study advocates for broader integration of VR technologies into medical curricula to enhance instruction quality and patient-centered care.

## Introduction

### Background

The domain of health care education is currently undergoing a monumental shift, facilitated by advancements in immersive technologies such as virtual reality (VR). Immersive technologies have demonstrated significant potential in transforming medical and health education, including enhanced training in surgical procedures [1,2], improved understanding of complex biomedical processes through immersive visualization [3,4], and more empathetic patient care through simulated patient interactions [5,6]. Despite its promise, VR technology faces challenges such as system fidelity and presence, which can impact user experience and learning outcomes [7]. Addressing these challenges is essential for the effective design and implementation of VR training modules.

Moreover, VR provides a safe and controlled environment for students to practice and make mistakes without direct consequences on actual patients, increasing their confidence and proficiency before real-world clinical scenarios [8,9]. Concurrent with this paradigm shift, immersive technologies like VR have emerged as an effective tool in the instruction of empathy among nursing students [10-12]. Empathy, being a fundamental aspect of the nursing profession, has been shown to improve patient adherence to treatment plans [13], satisfaction levels [14], and overall health outcomes [15]. Specifically, being immersed in virtual narratives allows students to navigate and process their own emotions, as well as respond appropriately within simulated scenarios [16]. This innovative approach offers a safe space for students to handle the emotional complexities associated with patient care, thus better preparing them for future clinical encounters involving nuanced emotional interactions.

VR simulation has also been studied in the context of nursing education [17-21]. In our recent study [22], we found that VR-based role-playing games enhanced cognitive empathy among nursing students. However, affective empathy remains underexplored in VR-based nursing education. Cognitive empathy involves understanding another’s perspective, whereas affective empathy refers to sharing and responding to another’s emotional states [23]. A recent meta-analysis revealed that VR has a significant effect on perspective-taking outcomes (cognitive empathy) but lacks impact on affective empathy [23]. Therefore, the objective of the current research is to investigate the potential of VR to influence affective empathy among nursing students by exploring narrative-related psychological factors.

Research on immersive media indicates that perceived self-location (ie, being there in a virtual environment) is a key mechanism that explains the impact of VR on empathy [24,25]. VR-based empathy training programs often contain a story with plots and characters to help users experience a situation first-hand [26]. They comprise both medium-based (ie, of the virtual environment) and message-based (ie, of the narrative) elements [27].To understand how VR might enhance affective empathy, we explore specific psychological mechanisms: perceived self-location and narrative transportation.

Perceived self-location refers to the sensation of “being there” in a virtual environment, enhancing users’ immersion and empathy [24,25], which we hypothesize will lead to greater empathy by allowing users to more deeply understand and share the feelings of virtual characters. Narrative transportation is the cognitive and emotional absorption into a story, where individuals lose awareness of their physical surroundings and form intense connections with the narrative and characters [28,29]. This deep absorption can lead to significant shifts in attitudes and empathy toward others [30]. Research has found that VR narratives lead to higher levels of transportation compared with traditional media, which in turn enhance empathetic responses [31]. Therefore, we hypothesize that VR will enhance narrative transportation, significantly impacting affective empathy.

A noteworthy characteristic of VR-based empathy training programs is their ability to feature multiple characters, thus allowing users to experience a narrative from varying character perspectives [32,33] The perspective-taking aspect significantly impacts users’ emotional engagement with the character [34]. Research has found that users are more likely to experience emotional engagement toward characters that are portrayed positively [33] and are similar to them [35]. Therefore, we hypothesize that character perspective will impact emotional engagement, with higher engagement when viewing from the clinician’s perspective compared with the parents’ perspective due to character-user similarity, subsequently influencing affective empathy.

Moreover, we propose that perceived self-location, narrative transportation, and emotional engagement will form a sequential mediation model to account for the effect of VR-based training. Previous research on video games has found that the feeling of “being there” in the game is a predictor of flow, an experience similar to narrative transportation [36,37]. A recent study obtained similar findings: spatial presence predicted narrative transportation in a VR storytelling experience [38]. Furthermore, existing work suggests that narrative transportation is associated with an increase in emotional responses [39,40]. Hence, we predict that perceived self-location, enhanced by VR, will foster narrative transportation, which will, in turn, promote emotional engagement. Emotional engagement will then lead to affective empathy.

### Hypotheses and Research Questions

Based on the literature review, the following hypotheses were proposed to explore the interconnected roles of perceived self-location, narrative transportation, and emotional engagement in enhancing affective empathy through VR interventions:

1. Hypothesis 1: VR conditions will enhance (1) perceived self-location and (2) narrative transportation compared with non-VR conditions.
2. Hypothesis 2: the participants will experience higher emotional engagement when viewing the narrative from a clinician’s perspective compared with a parent’s perspective.
3. Hypothesis 3: perceived self-location will positively predict narrative transportation in VR-based training programs.
4. Hypothesis 4: narrative transportation will positively predict emotional engagement within VR-based experiences.
5. Hypothesis 5: emotional engagement will positively predict affective empathy.

In addition to these hypotheses, we proposed the following research questions to explore potential interaction and indirect effects:

1. Research question 1: Are there interaction effects between the media platform (VR vs non-VR) and character perspective (clinician vs parents) on psychological factors such as perceived self-location, narrative transportation, and emotional engagement?
2. Research question 2: Do perceived self-location, narrative transportation, and emotional engagement mediate the relationship between the media platform and affective empathy?

## Methods

### Design

**Figure 1.**
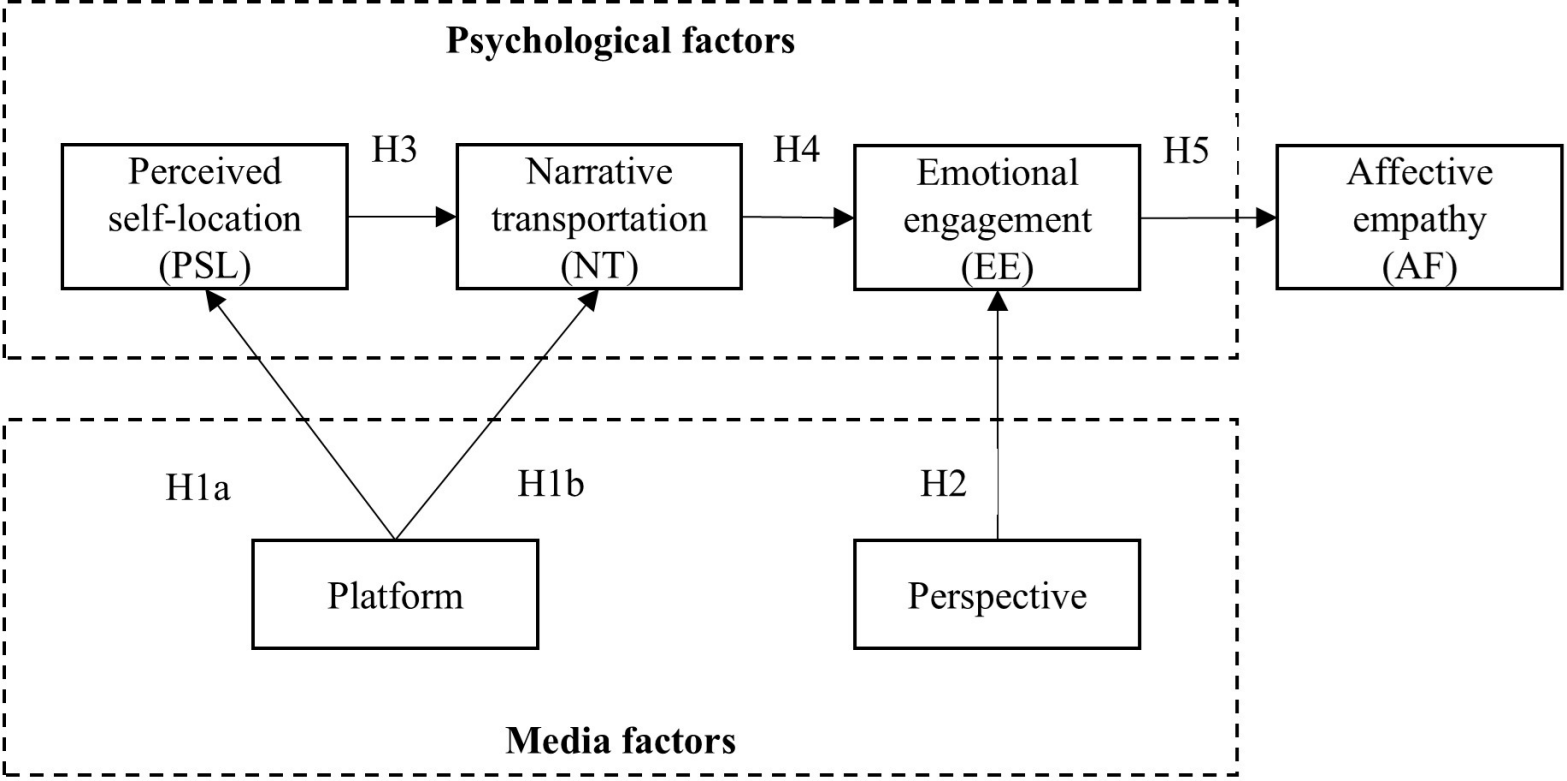
The proposed conceptual framework.

**Figure 2.**
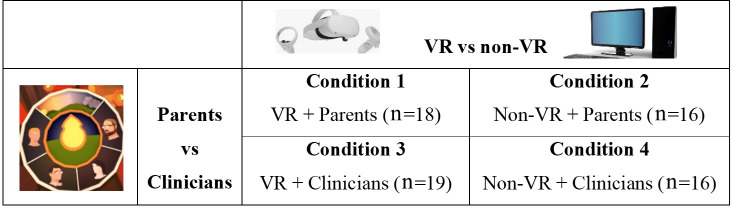
Four conditions of the study.

The proposed conceptual framework of this study is illustrated in Figure 1. This study used a 2×2 between-subjects experimental design to investigate the effects of the platform (VR vs non-VR) and perspective (parents vs clinicians) on nursing undergraduates’ empathy levels. Participants were randomly assigned to 1 of 4 conditions: VR parents, VR clinicians, non-VR parents, or non-VR clinicians. Figure 2 shows the 4 conditions of the study.

### Participants

A total of 69 nursing undergraduates from two Midwest universities participated in the study, predominantly female (57/69, 82.6%) and White (57/69, 82.6%), mostly juniors (39/69, 56.5%; sophomores: 24/69, 34.8%; seniors: 6/69, 8.7%). The average age was 22.13 (SD 3.70) years. The participants were recruited via university mailing lists and classroom announcements, with inclusion criteria requiring them to be nursing undergraduates aged 18 years or older. Most participants (54/69, 77.6%) reported they were not familiar with VR.

Before participation, all were screened for visual impairments and susceptibility to motion sickness; those with significant issues were excluded to avoid adverse effects during the VR experience.

Randomization was done through a drawing process, assigning participants to 1 of 4 conditions, ensuring balanced allocation between VR and non-VR platforms and the perspectives of parents or clinicians. Given the sample size and complexity of the 2×2 design, the study may be underpowered to detect small effects. No a priori power calculations were conducted; however, this exploratory research aims to investigate initial effects and generate hypotheses for future studies with larger samples.

### Experimental Procedures and Stimulus

Upon arrival at the research lab, participants provided informed consent and completed a short pretest questionnaire. They were then assigned to 1 of the 4 gameplay conditions as per the randomization process described above. The participants were informed that they could stop or report at any time if they experienced motion sickness or visual discomfort.

The participants engaged with the seventh chapter of the narrative-focused video game That Dragon, Cancer, titled “I’m Sorry Guys, It’s Not Good.” That Dragon, Cancer is an interactive narrative game developed by Numinous Games (Mainframe Studios) [41] that tells the real-life story of a family’s experience with their son’s terminal cancer diagnosis. The game is designed to evoke emotional responses and foster empathy through immersive storytelling [42]. This chapter was selected based on prior research demonstrating its efficacy in increasing empathy among medical students [42]. The gameplay allowed participants to experience a pivotal moment when Joel’s parents were informed of his terminal cancer diagnosis, navigating the scene from 4 unique perspectives: dad, mom, doctor, and nurse. The game is designed as a point-and-click adventure, which allows participants to trigger conversations and access a selected character’s inner thoughts.

The participants in the VR conditions used Oculus Go headsets, seated in a quiet room to minimize distractions. The headsets provided a 360-degree immersive experience with built-in headphones for audio. For the non-VR conditions, the participants used Dell laptops or iPads (Apple, Inc) with over-ear headphones, seated at a desk in the same room to ensure consistent environmental conditions. The game was presented on a standard screen, and the participants interacted using a mouse or touchscreen, replicating typical non-VR gameplay settings. The gameplay lasted approximately 10 minutes. The selection of this exposure time was based on previous studies indicating that brief VR experiences can effectively elicit emotional and empathetic responses [43]. Immediately after the gameplay, participants completed a posttest questionnaire assessing their empathy and gaming experience. This immediate administration was intended to capture their reactions and reduce potential recall bias.

### Instrument

Several validated scales were used to measure the constructs of interest. These measures are specifically applicable to our study’s context in nursing education and VR-based empathy training. First, the Spatial Presence Experience Scale, developed by Hartmann et al [44], was used to evaluate self-location and assess nursing students’ immersion in the simulated clinical scenarios. This validated scale, widely used in diverse media environments, measures 2 facets of the spatial presence experience: perceived self-location and potential actions, while also considering key factors that influence spatial presence. The study’s transportation scale was an adaptation from Green and Brock [28], which measures students’ absorption of patient stories that may foster empathy. In addition, the Emotional Engagement scale used in the study was sourced from Knol and Van Linge [45], which captures students’ emotional connection with virtual characters, vital for developing affective empathy. Affective empathy was assessed with 3 items from the validated empathy scale by Batson et al [46]. The participants responded to items on a 7-point Likert scale ranging from 1 (strongly disagree) to 7 (strongly agree). The results of these items were averaged to produce a composite score for data analysis. Covariates such as participants’ gender, age, race, and university affiliation were included in the following statistical analyses. Descriptive statistics, the items used, and reliability estimates for all scales are presented in Table 1.

**Table 1.** List of items used in the study and the descriptive statistics (N=69).

Variable	Statistics	Items
Perceived self-location [44]	Mean 5.19 (SD 1.12) Cronbach (α=0.88)	I felt like I was actually there in the environment of the presentation. It was as though my true location had shifted into the environment in the presentation. I felt as though I was physically present in the environment of the presentation. It seemed as though I actually took part in the action of the presentation
Transportation [28]	Mean 5.77 (SD 0.93) Cronbach α=0.83	I could picture myself in the scene of the events described in the story. I was mentally involved in the story while watching it. I wanted to learn how the story ended.
Emotional engagement [45]	Mean 5.83 (SD 0.98) Cronbach α=0.83	The story affected me emotionally. During the media experience, when the characters suffered in some way, I felt sad. I felt sorry for some of the characters in the story.
Affective empathy [46]	Mean 5.65 (SD 1.24) Cronbach α=0.93	Did watching/playing this video make you feel __ softhearted sympathetic compassionate

### Statistical Analysis

Data analysis was performed using SPSS 29 statistical software. Hypotheses H1, H2, and RQ1 were assessed through a series of analyses of covariance (ANCOVA), controlling for covariates such as participants’ gender, age, race, and university affiliation. Covariates such as participants’ gender, age, race, and university affiliation were included in the ANCOVA because these demographic factors have been shown to influence empathy levels and responses to VR experiences [23]. Including these covariates helps control for potential confounding variables, ensuring that the effects observed are attributable to the experimental manipulations rather than demographic differences. Hypotheses H3-H5 and RQ2 were tested with mediation analyses using the PROCESS macro, following a bootstrap estimation approach with 5000 samples, based on Hayes’ Process Model 6 [47]. Control variables were also included in these analyses to control for potential confounding influences.

### Ethical Considerations

This study was approved by the Institutional Review Board at Ball State University (approval number 1386023‐1). All participants provided informed consent before data collection. To acknowledge their participation, they received extra course credits as compensation. Confidentiality and privacy were maintained, and participants had the right to withdraw at any time without consequences.

## Results

### Descriptive Statistics and Correlation Analysis

The descriptive statistics for each condition, including mean and SD, are presented in Table 2. In addition, skewness and kurtosis values were assessed to check the normality of the data distribution, and the results were within the acceptable range confirming the appropriateness of the data for further analysis. A correlation analysis was conducted to identify any potential multicollinearity issues among the variables. The results indicated that while variables were correlated, they did not exceed the threshold that would suggest multicollinearity, thus ensuring the independence of predictors. The means for all variables were above the midpoint of the scale, indicating generally high levels of perceived self-location, narrative transportation, emotional engagement, and affective empathy among participants. The SD values ranged from 0.83 to 1.24, suggesting moderate variability in responses.

**Table 2.** Means and SEs by experimental conditions (N=69). Participants’ race, gender, school year, and university affiliation were controlled.

Platform and perspective		Perceived self-location			Narrativetransportation		Emotional engagement		Affective empathy	
**VR, mean (SE)**										
	Parents (n=18)	5.58 (1.07)			6 (0.87)		5.85 (0.72)		5.76 (1.43)	
	Clinicians (n=19)	5.66 (0.98)			5.81 (1.07)		6.02 (1.04)		5.84 (1.15)	
**Non-VR, mean (SE)**										
	Parents (n=16)	4.50 (1.34)			5.27 (1.16)		5.31 (1.26)		5.23 (1.41)	
	Clinicians (n=16)	4.89 (0.65)			5.96 (0.59)		6.13 (0.7)		5.75 (0.9)	

### Analysis of Covariance

The analysis of covariance (ANCOVA) included checks for assumptions such as homogeneity of variances, assessed using the Levene test. The results of these tests confirmed that the assumptions of ANCOVA were met across all variables of interest. Specifically, Levene test results for homogeneity of variances were *F*_3,65_=2.146, *P*=.10 for self-location; *F*_3,65_=1.566, *P*=.20 for narrative transportation; *F*_3,65_=0.904, *P*=.44 for emotional engagement; and *F*_3,65_=0.620, *P*=.60 for affective empathy. These results suggest that the variances of the residuals were not significantly different from each other across groups for each variable, thus fulfilling one of the key assumptions for conducting ANCOVA and lending validity to the subsequent analyses.

Based on our findings (Table S1 in Multimedia Appendix 1), the platform of the game had a significant influence on perceived self-location (*F*_1,61_=6.60, *P*=.01, partial η^2^=0.098), indicating that VR has a stronger influence on perceived self-location compared with non-VR environments. This substantial difference in perceived self-location depending on the platform used provided support for hypothesis H1a, suggesting VR’s unique capacity to enhance users’ sense of presence within the virtual environment. We also examined the effect of the adopted perspective on participants’ emotional engagement. The results revealed a significant effect (*F*_1,61_=4.76, *P*=.03, partial η^2^=0.072), indicating that participants who assumed the clinician’s perspective exhibited greater emotional engagement compared with those adopting a patient’s perspective. This finding supported hypothesis H2, highlighting the importance of perspective in influencing emotional responses in VR settings. However, the effect of VR on narrative transportation did not yield significant results (*F*_1,61_=0.90, *P*=.35, partial η^2^=0.014), thereby not supporting hypothesis H1b.

**Figure 3.**
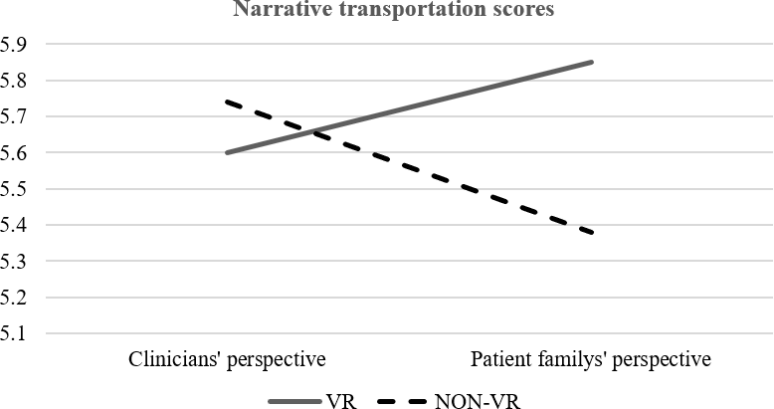
The interaction effect of media factors on psychological factors.

To answer the first research question, we also test whether there is an interaction effect of media factors on psychological factors. The results revealed that the platform and perspective had an interaction effect on narrative transportation (*F*_1,61_=4.68, *P*=.04, partial η^2^=0.070). The post hoc analysis indicated that participants experiencing the game from the perspective of patients’ families in a non-VR platform exhibited the lowest level of narrative transportation. This nuanced finding sheds light on how different combinations of platform and perspective can uniquely affect the immersive experience of users. These interaction effects are further elucidated in Figure 3, providing a visual representation of these dynamics. Specifically, the narrative transportation scores for clinicians and patient families under VR (solid line) and non-VR (dashed line) conditions showed diverging trends between the two perspectives.

### Mediation Analysis

The mediation analyses using Hayes’ PROCESS model 6 revealed several direct and indirect effects. Regarding direct effects, self-location was found to have a significant positive effect on transportation (*b*=0.54, *P*<.001). Transportation had a positive effect on emotional engagement (*b*=0.74, *P*<.001). Emotional engagement was found to have a strong positive effect on affective empathy (*b*=0.84, *P*<.001). These findings, supporting H3-H5, illustrate the psychological process from self-location in VR, through narrative transportation and emotional engagement, to the ultimate development of affective empathy. We used 5000 bootstrap samples to generate bias-corrected confidence intervals for the indirect effects. The significance of the mediation pathways was determined by examining whether the confidence intervals excluded zero. All variables were included in the model simultaneously, and control variables were accounted for in the analysis. The model fit was assessed, and all pathways were found to be significant, confirming the validity of the mediation pathways.

**Figure 4.**
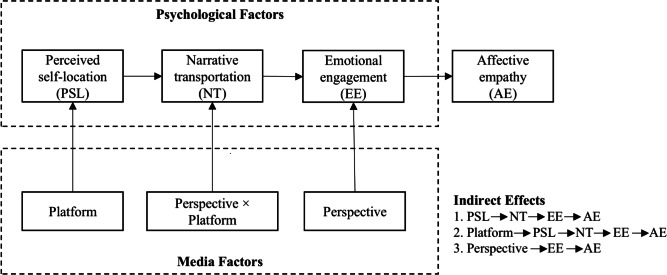
The revised conceptual framework.

The results also uncovered three sets of indirect effects, which answered our second research question. First, perceived self-location exerted an indirect effect on affective empathy through narrative transportation and emotional engagement (indirect effect=0.25, 95% CI 0.0610-0.5048). Second, the platform had an indirect effect on affective empathy through perceived self-location, narrative transportation, and emotional engagement (indirect effect=0.25, 95% CI 0.0610-0.5048). Finally, the perspective adopted by the participants was found to have an indirect effect on affective empathy through emotional engagement (indirect effect=0.27, 95% CI 0.0059-0.6153). These indirect effects, along with the direct effects, are represented in the revised conceptual framework presented in Figure 4.

## Discussion

### Summary and Interpretations of the Findings

The study investigates the impact of media platforms and players’ perspectives on perceived self-location, narrative transportation, emotional engagement, and affective empathy within medical education, which capture key psychological processes essential for developing empathy in clinical practice. The results indicate that all psychological factors were influenced by media elements, albeit through different mechanisms.

Our hypothesis H1a was supported, demonstrating that playing the game in VR (vs non-VR) significantly increased perceived self-location. This finding is consistent with prior studies [24,25]. However, contrary to our hypothesis H1b, VR (vs non-VR) did not differ in narrative transportation, which echoes findings from a recent meta-analysis [27]. This nonsignificant finding suggests that while VR enhances the sense of presence or self-location, it may not necessarily increase narrative transportation compared with non-VR platforms. One possible explanation is that narrative transportation is more strongly influenced by the quality of the narrative itself rather than the medium through which it is delivered [28]. It is possible that the narrative content was equally engaging in both VR and non-VR formats, resulting in similar levels of transportation. Future research could explore how different narrative structures or content types interact with VR to affect narrative transportation.

Regarding our hypothesis H2 adopting a clinician’s perspective during the VR experience significantly influenced emotional engagement, the result is consistent with previous research [32,33]. This insight emphasizes the value of learning from other clinicians in strengthening emotional ties with patients, thereby fostering affective empathy. The findings showed the potential of VR in medical education by enhancing perceived self-location, which can improve empathy toward patients. The significant influence of perspective adopted by players reiterates its role in enhancing emotional engagement, crucial for fostering empathy in health care professionals.

The proposed direct relationships between psychological factors and affective empathy (H3-H5) were all supported. The findings demonstrate that a sense of perceived self-location in VR can enhance narrative transportation, leading to increased emotional engagement. In turn, this fosters affective empathy, a critical skill for health care professionals to understand and respond to patients’ emotional experiences effectively, a critical skill for health care professionals to understand and respond to patients’ emotional experiences effectively. This sequential process confirms the potential of VR in facilitating immersive, emotionally engaging learning experiences in medical training, promoting the development of affective empathy.

The exploration of the research questions (RQ1 and RQ2) yielded compelling insights. The first research question investigates potential interaction effects. Our findings indicate a significant interaction effect among these conditions, specifically revealing that scores on narrative transportation were significantly lower in the condition using non-VR with a patient’s perspective compared with the other 3 conditions. This interaction effect suggests that the combination of platform and perspective plays a crucial role in influencing narrative transportation. One possible explanation is that adopting the patient’s perspective in a non-VR environment may not provide sufficient immersion or sensory cues to facilitate deep engagement with the narrative. In contrast, VR may compensate for the less immersive perspective by enhancing sensory immersion, while adopting a clinician’s perspective may align more closely with the students’ professional identity, facilitating engagement even in non-VR settings. This finding indicates that the effectiveness of narrative transportation may depend on the congruence between the medium, the perspective adopted, and the user’s own identity and experiences. Future studies could explore how personal relevance and role identification influence narrative engagement across different platforms.

For the second research question, the study uncovered 3 indirect effects: perceived self-location impacted affective empathy via narrative transportation and emotional engagement, the platform influenced affective empathy through self-location, narrative transportation, and emotional engagement, and the perspective affected affective empathy through emotional engagement.

### Theoretical Contributions and Practical Implications

This study makes significant theoretical contributions to the fields of empathy research and narrative communication. It demonstrates how VR can influence affective empathy through mechanisms such as perceived self-location, narrative transportation, and emotional engagement, thereby deepening our understanding of the integral role immersive technologies play in fostering critical emotional competencies. By identifying the sequential mediation of these psychological factors, our findings extend existing theories on empathy development and immersive media, providing empirical evidence within the context of nursing education.

These findings add valuable empirical evidence to empathy research and highlight the importance of immersive, technology-enabled experiences in shaping affective responses. Specifically, our study fills a gap in the literature by focusing on affective empathy rather than cognitive empathy, which has been less examined in VR research. Furthermore, by exploring the interaction between character perspective and affective empathy within VR environments, the study offers a novel perspective on empathy development and enriches narrative communication research. This contributes to a more nuanced understanding of how perspective-taking in VR can differentially impact emotional engagement and empathy outcomes, which has practical implications for designing effective educational interventions.

From a practical standpoint, the findings offer actionable insights for integrating VR into nursing education. We propose that nursing programs should incorporate VR experiences that emphasize perspective-taking from a clinician’s viewpoint to enhance emotional engagement and affective empathy among students. To address potential barriers such as cost, accessibility, and technological limitations, nursing programs could start by incorporating affordable VR solutions like stand-alone VR headsets or 360-degree video experiences, which are more feasible than high-end VR systems or simulation stations. For accessibility, it is important to ensure that VR experiences are also designed for students in the classroom settings. Technological limitations, such as a lack of technical expertise among faculty, can be mitigated through training workshops and technical support services. In addition, curricula should be designed to seamlessly integrate VR experiences into existing courses, perhaps starting with pilot programs to evaluate effectiveness before broader implementation. By proactively addressing these barriers, educators can more effectively leverage VR technology to enhance empathy training in nursing education. By implementing these recommendations, educators and institutions can leverage VR technology to significantly enhance the quality of medical education and training, especially in the domain of empathy development.

### Limitations and Future Research Directions

This study has several limitations. First, while the sample size was sufficient to yield statistical power, it was relatively small. The participants were primarily female, white nursing undergraduates from two Midwestern universities. The small sample size may have also limited our ability to detect smaller effect sizes. The homogeneity in gender and race may have influenced the results, as previous research suggests that empathy levels and responses to VR experiences can vary across different demographic groups. For instance, gender differences have been observed in emotional processing and empathetic responses, which could affect how participants engage with VR-based empathy training. Therefore, our sample may limit the generalizability of the results.

Future studies should prioritize recruitment strategies that enhance demographic diversity to ensure the broader applicability of findings. One approach is expanding outreach to institutions with more diverse student populations, such as historically Black colleges and universities, Hispanic-serving institutions, and community colleges. Establishing partnerships with nursing programs in urban and rural areas can also help reach a wider range of participants with different socioeconomic and educational backgrounds. In addition, leveraging professional nursing associations, student organizations, and social media platforms can improve the recruitment of participants from underrepresented groups. Providing flexible participation options, such as internet-based study components or varied scheduling, may further increase accessibility and encourage participation from nontraditional students, working professionals, or those with caregiving responsibilities. Implementing these strategies can enhance inclusivity and variability, ultimately strengthening the generalizability of future research.

Second, the research design offered only a brief, single-session exposure to VR and non-VR platforms and varying character perspectives. This short exposure duration may not have allowed sufficient time for participants to fully immerse themselves in the VR experience or for the effects on empathy to fully manifest. This limited interaction may not fully capture the potential effects of extended VR-based training on empathy. Longer or repeated exposures could provide a more accurate assessment of VR’s impact on empathy development. Therefore, longitudinal studies are recommended to investigate the long-term effects of VR on empathy, providing insights into the sustainability and potential long-term integration of VR into medical education.

Third, while we used self-reported scales that have been validated and widely used, these measures may introduce a certain level of response bias. Self-reported data can be influenced by social desirability or participants’ subjective interpretations of the questions, which may affect the accuracy of the results. Future investigations could stand to gain significantly from incorporating more objective evaluative methods, such as physiological and behavioral observations, that would serve to substantiate the self-reported data. For example, using biometric measures like heart rate variability or skin conductance could provide objective insights into emotional engagement and empathy responses. In addition, behavioral assessments during simulated interactions could offer tangible evidence of empathy development. Implementing mixed-method approaches would mitigate reliance on self-reports and enhance the validity of future research findings.

Finally, although the usability of the VR interface is important, our study did not assess usability using standard questionnaires such as the System Usability Scale [48] or the Usefulness, Satisfaction, and Ease of use [49] questionnaire. Future research could incorporate these usability measures to provide a more comprehensive evaluation of VR interfaces in educational settings. Usability testing plays a crucial role in ensuring that VR interventions in nursing education are intuitive, user-friendly, and meet the needs of learners. A system that is difficult to navigate or provides a poor user experience can disrupt engagement and limit the effectiveness of the intervention. Future research should integrate standardized usability assessments, such as the System Usability Scale or Usefulness, Satisfaction, and Ease of use questionnaire, to systematically evaluate the user experience. These measures will help identify areas for improvement, offering insights into how the VR interface aligns with learning goals and how usability affects student engagement and comprehension. By incorporating such evaluations, design improvements can be made—whether refining the interface, enhancing interaction features, or adjusting the VR experience to better suit diverse learning styles. Ultimately, addressing usability issues can improve the practical application of VR in nursing education, ensuring that immersive learning experiences are both accessible and impactful for real-world clinical practice.

### Conclusion

In this study, we explored the interactive roles of media platforms and perspective-taking in shaping key psychological factors, including perceived self-location, narrative transportation, emotional engagement, and affective empathy, in nursing education settings. The initial findings provide empirical evidence for the potential of immersive technologies as applicable pedagogical tools, particularly in teaching and training future health care providers. The capacity of virtual reality to facilitate the feeling of presence, build emotional engagement, and foster empathy among nursing students shows the potential to foster a greater degree of patient-centered care. Therefore, this study advocates for a wider consideration of integrating technologies into health care education curriculum design and development. The potential benefits and financial viability of VR technologies could enrich pedagogical experiences and pave the way for the emergence of competent health care professionals, well-equipped to navigate different scenarios in health care delivery.

## Supplementary material

10.2196/59083 Multimedia Appendix 1 Analysis of covariance (ANCOVA) results for multiple outcome measures.

## Abbreviations

ANCOVA: analysis of covariance
VR: virtual reality
