# Supplementary material for: Media-Induced and Psychological Factors That Foster Empathy Through Virtual Reality in Nursing Education: 2×2 Between-Subjects Experimental Study
Source: JMIR Med Educ. 2025 Mar 31;11:e59083. doi: 10.2196/59083 (PMC11975256; doi:10.2196/59083)
Supplement: Multimedia Appendix 1 [file mededu-v11-e59083-s001.docx]

| **Table S1.** | | | | | | |
| --- | --- | --- | --- | --- | --- | --- |
| Source | Variables | Sum of Squares | df | F value | P value | Partial η² |
| Platform | Affective Empathy | 0.054 | 1 | 0.042 | .838 | .001 |
|  | Transportation | 0.794 | 1 | 0.895 | .348 | .014 |
|  | Self-Location | 6.503 | 1 | 6.660 | .012* | .098 |
|  | Emotional Engagement | 0.736 | 1 | 0.820 | .369 | .013 |
| Perspective | Identification | 0.295 | 1 | 0.513 | .476 | .008 |
|  | Affective Empathy | 2.356 | 1 | 1.852 | .179 | .029 |
|  | Transportation | 1.049 | 1 | 1.182 | .281 | .019 |
|  | Self-Location | 0.996 | 1 | 1.020 | .316 | .016 |
|  | Emotional Engagement | 4.268 | 1 | 4.758 | .033* | .072 |
|  | Identification | 0.013 | 1 | 0.022 | .881 | <.001 |
| Platform × Perspective | Affective Empathy | 3.614 | 1 | 2.841 | .097 | .045 |
|  | Transportation | 4.091 | 1 | 4.608 | .036* | .070 |
|  | Self-Location | 0.395 | 1 | 0.404 | .527 | .007 |
|  | Emotional Engagement | 2.871 | 1 | 3.200 | .079 | .050 |
|  | Identification | 0.782 | 1 | 1.358 | .248 | .022 |
| Total | Affective Empathy | 2312.333 | 69 |  |  |  |
|  | Transportation | 2360.444 | 69 |  |  |  |
|  | Self-Location | 1945.563 | 69 |  |  |  |
|  | Emotional Engagement | 2415.333 | 69 |  |  |  |
|  | Identification | 2083.056 | 69 |  |  |  |
